# Is Depression Underdiagnosed by Oncologists? Implications from a Registry Study

**DOI:** 10.3390/cancers18030402

**Published:** 2026-01-27

**Authors:** Thomas Licht

**Affiliations:** 1 Ludwig Boltzmann Institute for Rehabilitation Research, Reizenpfenniggasse 1, 1140 Vienna, Austria; thomas.licht@lbg.ac.at; Tel.: +43-676-831275040; 2 Onkologisches Rehabilitationszentrum St. Veit im Pongau, St. Veiter Str. 48, 5621 St. Veit im Pongau, Austria

Patients with cancer often suffer from mental health problems in the face of a potentially life-threatening illness, particularly depression, adjustment disorders, and anxiety. However, the frequency of psychological distress has been determined and assessed quite differently in numerous studies. According to a systematic review analyzing studies prior to the onset of the COVID-19 pandemic, the prevalence of depressive symptoms among long-term cancer survivors ranges from 5.4% to 49% [1]. In a meta-analysis including 94 studies that utilized structured interviews with patients in oncological, hematological, and palliative care settings, the prevalence of all types of depression was determined as 24.6% [2]. Of those, 16.5% of cases were classified as major depression and 9.6% as minor depressive symptoms.

In “High Frequency of Depression in Advanced Cancer with Concomitant Comorbidities: A Registry Study,” by P. Strang and T. Schultz [3], which appeared in the section Updates on Depression among Cancer Patients of *Cancers*, the occurrence of depression in cancer patients’ last year of life is examined. The authors compared the socioeconomic characteristics, gender, and age of the patients, as well as risk factors such as comorbidities and frailty. This was a registry study conducted on a Swedish population, excluding only residents of nursing homes. The data was collected from an administrative database of medical records. Using a registry study design, the authors collected real-world data and reflected the actual situation in the population. In this study, the prevalence of depression was reported as 4.3%. Hence, the question arises as to what causes these substantial differences between previously published data on the frequency of depression in cancer patients and why the study conducted in Sweden identified a considerably lower prevalence in people with advanced tumor diseases.

A variety of methods and different criteria are used to diagnose depression. A systematic review noted 106 different methods of assessment in 202 articles on depression in palliative cancer patients [4]. The confusion might be even greater in daily oncologist practice than in controlled studies, as standardized psychological procedures are not routinely used due to time constraints.

In addition, the numbers of diagnoses of depression documented in medical records should be viewed with caution. In a clinical study, patients of non-psychiatric wards in community hospitals were interviewed by research psychiatrists using standardized techniques, and the findings were compared with the respective diagnoses established by the ward physicians [5]. Of those suffering from depression, only 45.7% were identified as mentally ill by non-psychiatric ward physicians. Moreover, only a minority of 21% of depressed patients received a psychiatric discharge diagnosis even though their depression had been recognized by the physicians. The authors concluded that depression was frequently overlooked in everyday clinical work and that routine discharge diagnoses provided only limited information about how often ward physicians recognized mental disorders.

It is conceivable that these factors may have a confounding influence on the frequency of diagnoses in registry studies such as the present Swedish study [3]. Registers of administrative data depend on what the treating physicians have recorded. The actual prevalence of depression among cancer patients in their final year of life may be considerably higher than 4.3%.

Hence, it is not straightforward to determine how many cancer survivors actually suffer from depressive symptoms or major depression, respectively. In terms of methodology, comprehensive clinical assessment by structured or semi-structured interviews such as the Composite International Diagnostic Interview (CIDI) or the Structured Clinical Interview for Diagnostic and Statistical Manual of Mental Disorders, Fifth Edition—Clinical Version (SCID-5-CV) are considered the gold standard for diagnosing depression [6,7]. However, these methods are time-consuming and require special knowledge and training.

To gain a clearer understanding of what we are talking about, it is worth reviewing the decisive criteria used for the diagnosis of depression. They are described in the *Diagnostic and Statistical Manual of Mental Disorders* (current version: fifth edition, text revision, DSM-5-TR) [7]. Briefly, at least one core symptom is mandatory for major depression: a depressed mood for most of the day or diminished interest or pleasure (anhedonia). Furthermore, any four of the following additional symptoms are required: changes in weight or appetite; sleep disturbances; psychomotor slowing or agitation; fatigue or loss of energy; feeling of worthlessness or excessive or inappropriate guilt; issues of concentration or decision making; or suicidal ideation. The symptoms should persist for more than two weeks.

The term subsyndromal depression, frequently referred to as minor depression, is used to refer to the presence of two to four symptoms for more than two weeks. The symptoms should not be directly related to another disease or illness. Otherwise, this would be referred to as a depressive disorder due to another medical condition. Furthermore, adjustment disorder is characterized by the development of emotional or behavioral symptoms as a response to a discernible stressor causing distress or significant impairment in functioning.

Alternatively, the World Health Organization’s definitions of ICD-11 can be used [8]. These utilize similar criteria, but there are some distinct differences, e.g., the inclusion of hopelessness as a separate criterion. Furthermore, the ICD-11 classifies depression caused by different underlying diseases as secondary mental or behavioral syndromes. In general, ICD-11 takes a more flexible approach, allowing clinicians to adapt symptoms to a diagnosis [9].

In clinical cancer treatment practice it is rarely possible to conduct detailed structured interviews due to a lack of resources. Instead, questionnaires are often employed to assess the patients’ psychological distress, particularly depression and anxiety. Among many validated instruments, the Hospital Anxiety and Depression Scale (HADS) [10], the Patient Health Questionnaire-9 (PHQ-9) [11], and Beck’s Depression Inventory (BDI-II) [12] are commonly used for the assessment of distress or the clinical diagnosis of depression, respectively, and for scientific studies in oncology. The inventories were compared with structured interviews in patients with heart disease. Thereby, the detection of moderate to severe depressive disorders by BDI-II and the HADS was excellent, while mild depressive disorders were only detected to a limited extent [13]. Furthermore, the Brief Edinburgh Depression Scale (BEDS) is a validated and sensitive screening instrument that uses six items [14].

On the other hand, cancer-specific questionnaires like the QLQ-C30 of the European Organization for Research and Treatment of Cancer (EORTC) or SCNS-SF34 are often used for assessment of the quality of life and needs of patients [15,16]. In these instruments, psychological distress is examined with some the questions, e.g., feeling depressed, irritable, or tense and whether the patients worried or experienced difficulties remembering things. Obviously, these instruments provide much less specific information about the presence of a depressive disorder. In conclusion, the choice of instrument is of foremost importance for the diagnosis of clinically relevant depression.

The prevalence of depression in a specific population—in this case cancer patients in their final year of life, as analyzed in the Swedish registry study [3]—should be considered in relation to the general population. A recent study used BDI-II to establish normative data on depression based on a representative sample of the general Swedish population [17]. This population was found to be minimally depressive. Notably, women were significantly more depressed than men, and higher education and age were inversely correlated with depression. Higher levels of depression for women are not only observed in Sweden; similar results have also been reported with BDI-II in the general population of the Netherlands, Czechia, and Mexico [18,19,20].

While these investigations focused on mean scores for depression, in another study the number of individuals with depressive symptoms at a given time point was analyzed. The prevalence of clinically significant depression in the general Swedish population was determined as 10.8%, with 5.2% experiencing major depression [21]. The authors also calculated how the figures would change if a stricter definition of major depression was applied. This resulted in a prevalence of 4.4% in the general Swedish population. Notably, comorbidity was associated with higher symptom severity and a lower health-related quality of life. In particular, comorbidity with anxiety was associated with greater symptom severity and a lower health-related quality of life.

According to a large study, the risk of being depressed is more than five times higher in cancer patients than in the general population [22]. In this investigation, patients with pancreatic, brain, or thyroid cancers demonstrated the highest prevalence, while prostate cancer or malignant melanoma were associated with the lowest levels of depressive symptoms. In contrast, the highest frequency of depressions in the study by P. Strang and T. Schulz was identified in hematological malignancies, followed by brain tumors and gynecological and lung cancer [3]. In fact, there are noticeable differences between the reports with respect to distress in various cancer entities.

We conducted a study with 4400 cancer survivors who were evaluated for psychological distress prior to admission to a rehabilitation measure. As determined by the HADS, the highest depression scores were identified among liver, lung, thyroid, and bladder cancer survivors, while survivors of testicular cancer had the lowest levels [23]. Notably, high levels of psychological distress with anxiety and depression were repeatedly observed in thyroid carcinoma patients, although their prognosis is generally favorable in comparison to other cancer entities, which can last many years after the end of treatment [22,23,24].

A systematic review of 210 studies, which used questionnaires, identified the prevalence of clinical depression as 21.2% (range from 7.9% to 32.4%), with highest levels in colorectal, hematologic, brain, gastric, and lung cancer [25]. Several risk factors for the development of depressive disorders have been established in cancer patients. They include a younger age, female gender, a past history of mood disorders, substance abuse or other psychiatric conditions, a lack of adequate social support, and a lower socioeconomic status [9]. The prevalence of depression is highest in the acute phase of the disease and decreases afterwards [26].

There are large differences between the prevalence of depression in the Swedish cohort study and data from carefully conducted analyses based on structured interviews or questionnaires. In fact, according to [3] the rate of people suffering from depression in the last year of their lives would be even lower than in the general population. However, this is probably not an error in the conduct of the study or the data analysis but is based on the medical reports on which the survey is based. The chosen method carries the risk of several confounding factors; for instance it does not include reports on the severity or length of depressive episodes, and there is insufficient data on pre-existing psychiatric conditions. Taken together, this suggests that depression is underdiagnosed in clinical practice.

A special focus should be dedicated to comorbidities. In a British study, women with breast cancer and one comorbid chronic condition, e.g., hypertension, had an increased risk of depression [27]. The likelihood of depression was further increased in a dose–response fashion with an increasing number of chronic conditions. The results of the Swedish registry study are consistent with these reports and demonstrate a significant increase in depression rates among patients with multimorbidities, especially among frail patients [3]. Such a relationship is also recognized in other non-cancerous chronic diseases. In these conditions as well, the risk of mental disorders including depression increases with the number of somatic diseases [28].

Ignoring or failing to treat depression has far-reaching consequences for the patients affected. Depression leads to a poorer quality of life and compromises patient outcomes [29] and causes more emergency unit visits and psychiatric consultations [3]. It is well-known that cancer patients with depression have a higher risk of mortality, which applies to depression both before and after the cancer diagnosis [30,31]. Depression is associated with cancer recurrence, all-cause mortality, and cancer-specific mortality in women with breast cancer [31]. One should, however, keep in mind that depression itself carries a relative risk of 1.6 for all-cause mortality, even in the absence of cancer or comparable diseases [32]. Therefore, it can sometimes be difficult to attribute the increased mortality rates to the malignant disease itself or to depression.

Various factors contribute to the risk of increased mortality in cancer patients. Depression is associated with a lack of adherence to oncologic treatments [33]. For instance, women with breast cancer adhere significantly less to adjuvant endocrine therapy in [34]. Cancer patients with depression were less likely to receive intensive treatment with intravenous infusions in the registry study but were more likely to receive specialized palliative care [3]. Furthermore, there is an elevated risk of suicidality in cancer survivors, with 14.3% reporting suicidal ideation [35]. A higher risk of suicide has been observed in men with prostate cancer [36], women with breast or gynecologic cancers [37], and head and neck cancer [38]. Oncologists should be aware that suicidal ideation is more common in adolescent and young adult cancer patients than among older adults [39].

While not observed in the Swedish study, there is good evidence that socioeconomic factors modify the relative risk of depression in cancer patients. Fewer comorbidities, a higher education level, greater socioeconomic status, and positive social supportive factors lower the mortality risk in older adult cancer patients with depression [40]. Some of these factors, particularly the management of comorbidities and social support factors, can be influenced by oncologists as healthcare providers.

What are the reasons for the underdiagnosis of depression in clinical practice? A study exploring physicians’ knowledge of depression symptoms concluded that this is likely related to problems associated with the oncology working environment [41]. According to this study, oncologists had a good basic knowledge of the main symptoms of depression and were positive about participating in screening programs.

Conversely, in an older study the correlation between the assessment of stress levels and social support by radiation oncologists with screening instruments was poor [42]. In a total cohort of 298 patients, radiation oncologists recognized the presence of severe distress in only 11 of 30 severely distressed patients. An accurate perception of distress was particularly low in patients with head and neck cancer and lung cancer, as well as in patients from lower social classes. Moreover, the recommendations for supportive counseling did not correlate with patients’ distress or the extent of perceived support but rather with progressive disease and less denial behavior.

The clinical practice guideline of the European Society of Clinical Oncology (ESMO) [9] states that all patients with cancer should be regularly screened and assessed for symptoms of depression, e.g., feeling down, depressed, or hopeless; having little interest or pleasure in doing things; and thoughts of suicide in all phases of illness. This should be achieved with validated screening tools on a regular basis. The guideline recommends BDI-II and the PHQ-9 within all clinical cancer settings and the BEDS within palliative care settings. Similarly, the guideline of the American Society of Clinical Oncology (ASCO) recommends that all patients with cancer and cancer survivors should be evaluated for symptoms of depression and anxiety at periodic intervals across the trajectory of care with the use of validated measures [43]. In addition, the ASCO recommends that patients over the age of 65 receiving chemotherapy should receive geriatric assessments with validated tools and be screened for depression with the Geriatric Depression Scale (GDS) [44]. However, the GDS should be used with some caution, as it might overestimate depression in older patients compared to assessments via structured interviews [45].

Depression causes a reduced quality of life, impedes oncologic treatment, and increases the mortality of affected patients. By a comparison of studies using structured interviews, data from a registry study based on medical records appear to underestimate the rate of depressive cancer patients. The diagnosis of depression is complicated by a wide variety of different instruments used for this purpose. Cancer survivors and patients receiving ongoing oncologic therapies should be regularly screened for depressive symptoms with the use of validated instruments.
